# A holistic approach to evaluating Parkinson’s disease, using the Delphi method: a linear evaluation index

**DOI:** 10.1590/0004-282X-ANP-2020-0579

**Published:** 2021-12-13

**Authors:** Marcos SERRANO-DUEÑAS, Luis MASABANDA, Maria-Rosario LUQUIN

**Affiliations:** 1 Pontificia Universidad Católica del Ecuador, Facultad de Medicina, Quito, Pichincha, Ecuador. Pontificia Universidad Católica del Ecuador Facultad de Medicina Quito Pichincha Ecuador; 2 Hospital Carlos Andrade Marín, Servicio de Neurología, Quito, Pichincha, Ecuador. Hospital Carlos Andrade Marín Servicio de Neurología Quito Pichincha Ecuador; 3 Clínica Universitaria de Navarra, Departamento de Neurología, Pamplona, Navarra, España. Clínica Universitaria de Navarra Departamento de Neurología Pamplona Navarra España

**Keywords:** Parkinson Disease, Holistic Health, Delphi Technique, Psychometrics, Enfermedad de Parkinson, Salud Holística, Técnica Delfos, Psicometría

## Abstract

**Background:**

Parkinson’s disease (PD) is a chronic disease that presents a multitude of symptoms, with symptoms of both motor and nonmotor nature. The Delphi method is widely used to create consensuses among experts in a field of knowledge.

**Objective:**

In order to reach a consensus on the values that should be assigned to the different motor and nonmotor manifestations of Parkinson’s disease, a linear evaluation index (LEI) was created. Subsequently, the metric properties of this index were studied.

**Methods:**

120 consecutive patients with a Parkinson’s diagnosis were chosen in accordance with the UKPDSBB criteria. The Delphi method was used to reach a consensus among experts regarding the values of each of the manifestations included. Subsequently, the following attributes were analyzed: quality and acceptability of the data; reliability, in terms of internal consistency, reliability index, Cronbach’s alpha and standard error of measurement; and validity, in terms of convergent validity and validity for known groups.

**Results:**

Twenty-five experts participated. The importance factor did not differ between the first round and the second round (chi-square test). We analyzed the responses that assigned percentage values to the 10 dimensions of the LEI. Both in the first and in the second round, the values of the scattering coefficient Vr were always close to 0. The homogeneity index was 0.36; the corrected-item total correlation values ranged from 0.02 to 0.7; Cronbach’s α was 0.69; and the SEM was 4.23 (55.1%).

**Conclusions:**

The LEI was obtained through rigorous recommended methodology. The results showed adequate metric properties.

## RESUMO

**Antecedentes:**

La enfermedad de Parkinson (EP) es una enfermedad crónica que presenta una multitud de síntomas, tanto de naturaleza motora cuanto no motora. El método Delphi se utiliza ampliamente para crear un consenso entre expertos de un campo del conocimiento.

**Objetivos:**

Con el fin de llegar a un consenso sobre los valores que deben asignarse a las diferentes manifestaciones motoras y no motoras de la enfermedad de Parkinson, se creó el “Índice de Evaluación Lineal” (*linear evaluation index -* LEI). Posteriormente, se estudiaron las propiedades métricas de este índice.

**Métodos:**

Se eligieron 120 pacientes consecutivos con diagnóstico de Parkinson según los criterios del UKPDSBB. Se utilizó el método Delfos para llegar a un consenso entre los expertos sobre los valores de cada una de las manifestaciones incluidas. Posteriormente, se analizaron los siguientes atributos: Calidad y aceptabilidad de los datos. Fiabilidad: consistencia interna, índice de fiabilidad, alfa de Cronbach y error estándar de medida. Finalmente, Validez: validez convergente y validez para grupos conocidos.

**Resultados:**

Participaron 25 expertos, el factor de importancia entre la primera y la segunda rondas (prueba chi-cuadrado), no fue diferente. Analizamos las respuestas que asignaron valores porcentuales a las 10 dimensiones del LEI; tanto en la primera como en la segunda rondas, los valores del coeficiente de dispersión Vr siempre estuvieron cerca de 0. El índice de homogeneidad fue de 0,36; los valores corregidos de correlación ítem-total variaron de 0,02 a 0,7; alpha de Cronbach fue de 0,69. El SEM fue 4,23 (55,1%).

**Conclusiones:**

El LEI se ha obtenido siguiendo una rigurosa metodología recomendada. Los resultados han mostrado propiedades métricas adecuadas.

## INTRODUCTION

Parkinson’s disease (PD) is the second most common neurodegenerative disease in the world and affects between 2 and 3% of people over the age of 65^1^. The symptoms of PD include both motor and nonmotor symptoms. Up to 98% of patients have nonmotor symptomatology over the course of the disease, and these symptoms have a negative impact on patients’ quality of life^2^.

Some of the symptoms of PD are evaluated through subjective measurements, either directly, by the doctor, or indirectly, through the patient’s main caregiver^3^.

There is increasing evidence that both motor and nonmotor manifestations of PD are heterogeneous. This has led researchers to establish several nonmotor phenotypes for the disease, which is indicative that when evaluating PD, both motor and nonmotor symptoms should be considered^4^.

Once an overall assessment of patients with PD has been carried out, their physicians need to gauge which manifestations of the disease affect these patients the most and weight the symptoms accordingly. For example, physicians should ask themselves how seriously manifestations such as psychosis, depression and dysautonomia affect their patients, and how important bradykinesia and motor symptoms are in general.

The Delphi method (DM) is widely used to create a consensus among experts in a specific field of knowledge^5^. One crucial characteristic of this method is the anonymity of the experts involved, which allows experts to express their points of view freely, without retribution. Thus, the value assigned to each of a patient’s symptoms will be considered in terms of the importance of the symptom and not the merit of who proposes this.

Given that the researchers can view the criteria created by other experts in the group, they can reconsider their point of view. This generates a controlled feedback loop that gives researchers the opportunity to change their minds. Finally, a value is assigned to the answers, and these values can be statistically analyzed and interpreted^6^.

The Delphi method has been used to reach a consensus for making diagnoses of diseases such as progressive supranuclear palsy^7^ and advanced PD^8^.

We designed a cross-sectional study with the aim of reaching a consensus on the values that should be assigned to the different motor and nonmotor manifestations of PD. After data values had been collected, they were placed in a linear evaluation index (LEI), to evaluate patients holistically and study the resulting metric properties.

## METHODS

### Delphi panel

We followed the guidelines and suggestions that have been proposed for the Delphi method^5^^,^^6^. First, 30 renowned experts in the field of movement disorders and more specifically in PD were invited to participate in an online survey via e-mail. Five of them chose not to participate in the investigation: two because of conflicts of interest, two because of personal problems and one without stating a reason.

To be considered an expert, the participants needed to have achieved recognition in the field of movement disorders through having papers published in indexed journals within this field; and through having worked on movement disorders (i) in practice in a general hospital or university hospital; or (ii) in practice in a referral hospital; or (iii) in a national epidemiology/public health institution^9^.

An email was sent to the experts inviting them to take a survey. There were two sections in the survey. In the first section, the experts were asked to assign a level of importance (between 0=not important and 4=essential) for each of the following 10 dimensions involved in PD. 1) age dimension (AD); 2) motor dimension (MD); 3) depression dimension (DD); 4) anxiety dimension (AxD); 5) cognitive dimension (CD); 6) apathy dimension (ApD); 7) fatigue dimension (FD); 8) nonmotor dimension (NMD); 9) psychosis dimension (PsD); and 10) sleep dimension (SD). In the second part of the survey, the experts were asked to assign a percentage value to each of the dimensions, so that the final sum resulted in 100.

Twenty-three of the 25 experts responded within the first 72 hours. After a week without a response from the remaining two experts, the survey was resent to them. Both of these two remaining experts completed the survey within the subsequent 48 hours of receiving it.

Once we had collected the data from the survey, we proceeded to analyze the responses. The proportions of each of the values assigned to the dimensions from the first section of the survey (where the experts had to give a level of importance to each of the dimensions) were collected. From the second section of the survey (where a percentage was given to the dimensions), the median, mean, standard deviation and interquartile range were collected^10^. After the results had been analyzed, the experts were given the same survey again. This time, however, they were presented with their initial responses and the responses of the other experts and were given the choice to either change the values that they had given to these dimensions in the first round of the survey or not change them. All the experts responded within the following week.

### Patients

To calculate the sample size, the parameters suggested by Beavers et al.^11^ were applied. The UKPDSBB clinical diagnostic criteria^12^ were used to select the 120 PD patients who participated in the study. All the patients were treated in the Neurology Service of the Carlos Andrade Marín Hospital in Quito, Ecuador.

All the patients gave their informed consent to participate in the study, which was approved by the Teaching and Research Department of the Carlos Andrade Marín Hospital and by the Bioethics Committee of the University of Navarra (Spain).

The exclusion criteria consisted of the presence of any neurological disorder that caused disability: hemiplegia, blindness or deafness; or the presence of a serious acute illness.

All the patients were evaluated in the “ON” period. Demographic data of interest were collected, including age for AD. In addition, all of them were examined by means of the following tools: SPES-SCOPA^13^ to evaluate MD; HADS^14^ to analyze the presence of DD and AxD; PD-CRS^15^ to identify CD; AS^16^ to evaluate ApD; D-FIS^17^ to ascertain FD; SCOPA-PC^18^ to investigate PsD; and SCOPA-SLEEP^19^ for SD disorders. Lastly, using the NMSS^20^, the rest of the elements of the NMD were evaluated (except for depression, anxiety, apathy, fatigue, cognition, psychosis and sleep).

Apart from the rating scales indicated above, PIMS and CISI-PD were used to assess the quality of life and clinical status. PIMS is a 10-item, 4-domain scale. Its items are scored from 0 (no change) to 4 (severe), and the total scores for the scale range from 0 to 40. Lower scores indicate less impact from PD. PIMS has been recommended for use in PD^21^. CISI-PD assesses four domains: motor signs, disability, motor complications and cognitive status. Each domain is scored from 0 (normal) to 6 (severely compromised). The sum of these scores provides an overall evaluation index^22^.

The stages of the disease were evaluated using the Hoehn and Yahr (H&Y) scale^23^. Schwab and England (S&E scale)^24^ was used to study activities of daily living.

In addition to generating descriptive statistics of interest, the following factors were analyzed and parameters for them were defined:

- Data quality and acceptability: (i) lost data needed to not exceed 5%; (ii) the difference between the average and median needed to not exceed 10% of the highest possible score; and (iii) the floor and ceiling effects needed to not exceed 15%^25^
- Reliability: (i) internal consistency: the homogeneity index of the items needed to be ≥0.3^26^; (ii) reliability index: Cronbach’s alpha value needed to be greater than 0.7^27^; and (iii) the standard error of measurement (SEM) was obtained: the SEM needed to be equal to the standard deviation, multiplied by the square root of (1 minus Cronbach’s alpha), i.e. (StD * √1-reliability coefficient)^28^.
- Validity: (i) convergent validity. For this, the Spearman’s correlation coefficient (*rho*S) and the values suggested by Akoglu^29^ were used (0=no correlation; 0.1-0.3=weak correlation; 0.4-0.6=moderate correlation; 0.7-0.9=strong correlation; and 1=perfect correlation); and (ii) validity for known groups, for which we used the H&Y stages as a segmentation variable; values ≤0.05 were accepted as significant.

#### Statistical analysis of Delphi method

The data from the first round of the survey (the level of importance) were compared using the chi-square test. For the second round of the survey, in which the experts decided on what percentage to give for each of the dimensions, the dispersion coefficient V_r_ was gathered from both rounds. The dispersion coefficient V_r_ needed to have a value between 0 and 1, such that the closer it was to 0, the greater the degree of agreement between the experts also was^10^.

Lastly, the scores for the second round were multiplied by the mean value of the importance factor (obtained between the first and second rounds, which turned out to be the same). The sum of these scores resulted in a value of 105.6 (this number was then made equal to 100, to obtain the final value by means of the simple rule of three). For example, the motor dimension score was 30.3, multiplied by the importance factor, which was 1.2, resulting in a value of 36.36 (Equation 1).

105.6=100

36.36=X (1)

X=36.36*100/105.36

X=34.8

To obtain the LEI scores, the values for the level of importance for each dimension, from the second round of the survey, were multiplied by the average values of the percentages given by the experts. This determined a maximum final value of 105.6. Again, the rule of three allowed us to reach a value of 100. For example, if the score for the motor dimension in the second section of the survey was 30.3 and the level of importance from the first section of the survey was 1.2, the result would be 36.36 out of 105.6 which would therefore be 34.8 out of 100.

Continuing with the example of the motor dimension score, the original results had an average value of 28.1 (the maximum for the scale was 75), which yielded 13.03. Since 75 points was the maximum, 100% would be worth the maximum of 34.8 points. With CD, we proceeded by reversing the rule of three, since the higher the score was, the greater the cognition also was.

The maximum theoretical values attainable for each of the dimensions were as follows: the AD was arbitrarily determined at a maximum of 100 years old; MD, 75; DD, 21; AxD, 21; CD 134 (the minimum value in this study was 16); ApD 42; FD 32; NMD 168; PsD 21; and SD 33.

## RESULTS

Forty-seven (39.2%) of the patients included were women, with a mean age of 68.5 years and a disease duration of 9 years. The average dose of levodopa was 683.5 mg/day. Seventy-four patients (61.7%) were in stage III of H&Y. Fifteen patients (12.5%) were full-time employees and 73 (60.81%) were retired. The patients’ characteristics are shown in Table 1.

**Table 1.** Description of the sample (n=120).

	Median	Mean±SD	IQR	S	K
Number of years of schooling	7	9.6±5.2	8	0.5	-0.9
Number of years of disease	8	9±5.6	7	1.4	3.3
Number of years with L-Dopa	6	7.5±5.3	6.3	1.3	2.7
Dose (mg/day) of L-Dopa	750	683.5±225.5	250	-0.1	0.5
PIMS	21	19.9±7	10	-0.5	-0.1
CISI 1	3	3.3±0.9	1	0.05	-0.02
CISI 2	3	3.3±0.9	1	0.1	0.1
CISI 3	2	1.8±1.6	6	0.3	-1.1
CISI 4	2	2.1±1.2	2	-0.05	-0.9
Total CISI	10	10.1±4.1	6	0.3	-0.3

SD: standard deviation; IQR: interquartile range; S: skewness; K: kurtosis; PIMS: Parkinson’s Impact Scale; CISI: Clinical Impression of Severity Index for Parkinson’s disease.

The main results from the Delphi study were the following: twenty-five experts responded, and when we compared the factor of importance between the first round and the second round (using the chi-square test), we did not find any significant differences. When we analyzed the answers regarding percentage values for the 10 dimensions, the V_r_ dispersion coefficient values were always found to be close to 0 in the first round, and they were lower in the second round (Table 2).

**Table 2.** Assignment of values and importance factor by experts.

	First round	Second round	Dispersion coefficient v_r_		Importance factor		P-value ≤ comparison of the importance factor between rounds (chi-square)	Maximum final value
			First round	Second round	First round	Second round		
Age dimension	7.1	10.1	0.2	0.2	1.0	1.0	1.0	10.2
Motor dimension	32.1	30.3	0.3	0.2	1.2	1.2	0.4	34.8
Depression dimension	8.5	11.6	0.1	0.1	1.0	1.0	0.9	11.2
Anxiety dimension	4.5	7.1	0.2	0.2	0.8	0.8	0.9	6.1
Cognition dimension	5.9	9.7	0.3	0.1	1.0	1.0	0.9	9.6
Apathy dimension	5.5	6.0	0.5	0.2	0.9	0.9	0.9	5.3
Fatigue dimension	5.5	5.0	0.2	0.2	0.8	0.8	0.9	4.2
Nonmotor dimension	10.9	6.8	0.2	0.2	0.9	0.9	0.9	6.5
Psychosis dimension	9.7	5.8	0.3	0.3	0.9	0.9	0.9	5.3
Sleep dimension	9.7	7.1	0.2	0.1	1.0	1.0	0.8	6.8

The score from the 2^nd^ round was multiplied by the factor, which gave a total of 105.6; this amount was then made equal to 100, to obtain the final value by means of the simple rule of three (e.g. motor is 30.3 x 1.2=36.36; 36.36 is to 105.6 proportionally the same as 34.8 is to 100).

The homogeneity index was 0.36; the corrected-item total correlation values ranged from 0.02 to 0.7; Cronbach’s α was 0.69, with minimum and maximum values of between 0.39 and 0.63; and the SEM was 4.23 (55.1%).

Through integrating the LEI, the distribution of the data was revealed to be normal (Table 3). We found that the PsD presented a floor effect of 39.16%. All other dimensions had values within the requirements (Table 4).

**Table 3.** Description of the variables that made up the linear evaluation index.

	Average crude scores±SD	Median	Mean±SD	IQR	S	K
Age dimension	68.6±11	7.1	6.9±1.1	1.5	-0.5	0.1
Motor dimension	28.1±10.3	12.1	13±4.8	6.1	0.5	0.3
Depression dimension	5.4±2.7	2.7	2.8±1.4	2.1	0.4	-0.2
Anxiety dimension	6.4±3.6	1.7	1.8±1	1.4	0.1	-0.8
Cognition dimension	63.3±18.9	4.7	4.5±1.3	2.3	0.2	-0.6
Apathy dimension	12.5±8.9	1.5	1.5±1.1	2	0.1	-1
Fatigue dimension	9.6±6.6	1.2	1.2±0.8	1.3	0.6	0.2
Nonmotor dimension	31.6±18.7	1.1	1.2±0.7	0.8	1.4	3.7
Psychosis dimension	1.5±1.6	0.3	0.3±0.4	0.8	1.4	1.9
Sleep dimension	7.6±4.6	1.4	1.5±0.9	1.5	0.3	-0.7
Total		34.6	35.2±7.6	10.2	0.2	-0.2

SD: standard deviation; IQR: interquartile range; S: skewness; K: kurtosis.

**Table 4.** Metric properties of the variables that made up the linear evaluation index (LEI).

	Median	Mean	Mean-median difference	10% of total	Theoretical maximum	Floor effect	Ceiling effect
Age dimension	7.1	6.9	0.2	0.10	10.2	0.83	0.83
Motor dimension	12.1	13	0.9	3.48	34.8	0.83	0.83
Depression dimension	2.7	2.8	0.1	1.12	11.2	0.83	3.33
Anxiety dimension	1.7	1.8	0.1	0.61	6.1	4.16	1.66
Cognition dimension	4.7	4.5	0.2	0.96	9.6	0.83	0.83
Apathy dimension	1.5	1.5	0	0.53	5.3	8.33	0.83
Fatigue dimension	1.2	1.2	0	0.42	4.2	8.33	0.83
Nonmotor dimension	1.1	1.2	0.1	0.65	6.5	1.66	0.83
Psychosis dimension	0.3	0.3	0	0.53	5.3	39.16	1.66
Sleep dimension	1.4	1.5	0.1	0.68	6.8	4.16	0.83
Total	34.6	35.2	0.6	10	100	0.83	0.83

When we analyzed the convergent validity of the total LEI and its 10 dimensions, we found that the total LEI reached values of 0.66, compared with the PIMS; 0.74, compared with the CISI-PD; and 0.83, compared with the MD. Furthermore, there were values of -0.01 and -0.04 in relation to the DD and the PsD, respectively, with regard to the number of years of illness (Table 5).

**Table 5.** Convergent validity (Spearman’s correlation coefficient *rho*S).

	PIMS	CISI1	CISI2	CISI3	CISI4	Total CISI	Number of years of schooling	Number of years of disease	Number of years with L-Dopa	Dose (mg/day) L-Dopa	S&E
Age dimension	0.08	0.21	0.25	-0.09	0.42	0.21	-0.20	0.13	0.17	0.40	-0.24
Motor dimension	0.56	0.76	0.72	0.71	0.50	0.83	-0.19	0.52	0.59	0.50	-0.37
Depression dimension	0.53	0.30	0.40	0.23	0.30	0.37	-0.31	-0.01	0.06	0.16	-0.43
Anxiety dimension	0.49	0.35	0.39	0.21	0.24	0.35	-0.27	-0.04	0.05	0.04	-0.42
Cognition dimension	-0.41	-0.45	-0.49	-0.26	-0.82	-0.60	0.59	-0.19	-0.25	-0.39	0.57
Apathy dimension	0.41	0.40	0.51	0.21	0.68	0.53	-0.47	0.17	0.26	0.36	-0.6
Fatigue dimension	0.45	0.43	0.47	0.18	0.58	0.36	-0.32	0.09	0.19	0.24	-0.56
Nonmotor dimension	0.48	0.31	0.38	0.22	0.31	0.48	-0.12	0.15	0.22	0.33	-0.45
Psychosis dimension	0.25	0.23	0.25	0.09	0.23	0.22	-0.13	-0.04	0.05	0.08	-0.29
Sleep dimension	0.33	0.32	033	0.13	0.37	0.35	-0.20	0.26	0.32	0.36	-0.33
Total	0.66	0.69	0.73	0.52	0.52	0.74	-0.26	0.37	0.46	0.46	-0.8

PIMS: Parkinson’s Impact Scale; CISI: Clinical Impression of Severity Index for Parkinson’s disease.

In investigating the validity, we found that except for the PsD, all other dimensions and the total were significantly different.

## DISCUSSION

From the results regarding the Delphi consensus, it can be seen that all the experts gave a similar level of importance to each of the dimensions, so there were no variations between the first and second round (chi-square test, Table 2). In weighting the level of importance of each dimension, the V_r_ presented adequate values, of close to 0. Therefore, the participating experts assigned very similar weights to each of the dimensions. The V_r_ of the second round improved, thus resulting in a higher consensus being reached (Table 2). In summary, the experts considered that the ten dimensions included in the LEI were significant and assigned very closely matched levels of importance to them.

The dimension that was given the most weight in the LEI by the experts was that of the MD (34.8%), and the AD was next (10.2%). The remaining percentages (totaling 55%) corresponded to dimensions that were considered to be nonmotor. Thus, in this study, there was full incorporation of nonmotor dimensions, with the impact that they have on patients’ ability to function and quality of life^30^.

The quality of the data was adequate, such that 100% of the data collected could be computed. The demographic data describing the sample had characteristics of normality: minimal difference between the median and average, and values for asymmetry and kurtosis coefficients that were within the limit established (-1 to 1) for most of the variables^25^^,^^26^ (Table 1).

Regarding the dimensions that make up the LEI, their descriptions revealed that both the difference between the median and average, and the coefficients of asymmetry and kurtosis were within acceptable values, except for the kurtosis of the NMD and PsD, with values of 3.7 and 1.9, respectively (Table 3).

In analyzing the viability and acceptability of the scale, we found that the PsD contradicted the norm that the floor and ceiling effects would need to be less than 15%, such that a floor effect of 39.1% was reached. Our research used the same evaluation tool as used by Visser et al.^18^; they found that 78.7% of their subjects did not have problems or had only slight presence of psychosis (Table 4).

The homogeneity index reached a value of 0.36; the standard was ≥0.30; and the corrected-item total correlation values were adequate.

Although the alpha value obtained was 0.69, i.e. it did not reach the desired threshold of 0.7, two points should be considered. First, the alpha value is highly influenced by the number of items, as can be seen in its formula (the LEI only has 10 items) (Equation 2).

$$aC=\frac{N*R}{\left(\left[N-1\right]*R+1\right)}$$ (2)

Where: R is the mean of all the correlations and N is the number of items on the scale or questionnaire.

Secondly, as pointed out by Streiner^31^, the values initially required for alpha were between 0.5 and 0.6. We consider that although the theoretical target value of 0.7 was missed by one hundredth, the internal consistency of the data was good.

The value for the SEM needs to be 50% of the standard deviation (StD), for which accuracy above 75% is proposed^32^. We calculated a value of 4.2, which was equivalent to 55.1% of the StD.

**Figure 1.**
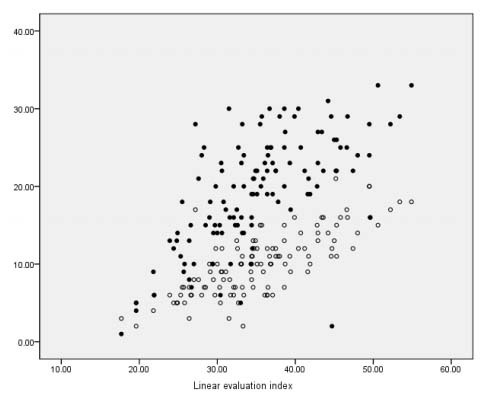
Scatterplot: linear evaluation index again PIMS (solid circles) and CISI-PD (empty circles).

The convergent validity of the LEI (sum total) showed a strong correlation, thus: S&E escalation (*rho*S -0.8); total CISI-PD (*rho*S 0.74) and total PIMS (*rho*S 0.66) (Table 5, Figure 1).

Through using the same analysis for each dimension, we compared the results with those for quality of life, which was evaluated using PIMS; with the total CISI-PD; and against the S&E scale. We found that the AD had a weak correlation: a low correlation with PIMS (*rho*S 0.08), which was similar to what had previously been reported^33^.

The MD (motor dimension) had moderate to strong correlations with the total CISI-PD (*rho*S 0.83). The DD (depression dimension) had moderate correlations, except for a very low one with the number of years of illness (*rho*S -0.01). The latter, we believe, may have been because depression and anxiety can precede the onset of Parkinson’s disease. Due to high prevalence, there is one report of 52.1%, although the sample in that study was a set of PD patients who underwent DBS^34^. The AxD (anxiety dimension) had correlations similar to those for depression, and a weak correlation with the number of years of illness (*rho*S -0.04). In the same study referred to above, it was also found that anxiety could precede PD. In that sample, anxiety had a prevalence of 55.5%.

The variable of the number of years of disease generally had weak correlations with the rest of the dimensions, except with MD (*rho*S 0.52). This may have been because, in our cohort, the patients had rather few years of disease (9±5.6 years). It has been shown in the literature that the greater the number of years of illness is, the greater the cognitive impediment will be^15^.

The ApD had a moderate correlation with the quality of life (*rho*S0.41). This level of correlation was slightly lower than what was obtained in other studies: *rho*S 0.56^20^ and *rho*S 0.51^35^.

The FD had a moderate correlation value (*rho*S 0.45), compared with the PIMS, and this was lower than what was gathered in another study in which the same evaluation tools were used (*rho*S 0.67)^36^.

The NMD had moderate correlations with the PIMS (*rho*S 0.48), total CISI-PD (*rho*S 0.48) and S&E scale (*rho*S -0.45). Lastly, the PsD had weak correlations with the other variables of interest. Previously, it has been reported that the patients’ age and number of years of illness, and the presence of dementia, were similar in patients with PD, both with and without psychosis^37^.

The SD reached correlations similar to what was reported in other studies, compared with the total CISI-PD (*rho*S 0.35), number of years of disease (*rho*S 0.26), number of years of levodopa (*rho*S 0.32) and dose of levodopa (*rho*S 0.36), which in the previous studies were *rho*S 0.39, 0.16, 0.18 and 0.22, respectively^38^.

An expert is an informed, specialized and knowledgeable individual in the specific field. To select experts, we followed the suggestions proposed by Pawlowski et al.^39^ and Robinson et al.^40^.

The final panel, which was composed of a group of heterogeneous experts, granted more credibility to the process than a homogeneous panel. This is because in a heterogeneous group there is a greater range of perspectives, which results in a more comprehensive study of the matter.

The dimensions included in the LEI were those that have consistently been reported as having the greatest impact on the quality of life of patients with PD^30^. In addition to this, our study included known nonmotor symptoms, which are often not reported by patients.

One of the limitations of the present study was the relatively small size of the sample of patients, as only 120 were studied. Another limitation was that all the patients came from the same specialized medical facility for patients with Parkinson’s, which is a national reference hospital.

The LEI now constitutes a tool that enables investigations during clinical consultations, without any sophisticated equipment, to provide comprehensive and objective evaluations on patients with Parkinson’s disease. Therefore, it provides overall information of enormous importance for decision-making.

In conclusion, the LEI was obtained through rigorous recommended methodology. The results showed that it has adequate metric properties, despite not having achieved the ideal value for Cronbach’s alpha. It is therefore a tool that has structural validity.
